# Biomechanical analysis of lifting on stable versus unstable surfaces—a laboratory-based proof-of-concept study

**DOI:** 10.1186/s40814-022-01157-2

**Published:** 2022-09-08

**Authors:** Wilhelmus Johannes Andreas Grooten, Edwin Billsten, Sebastian von Stedingk, Mikael Reimeringer

**Affiliations:** 1 Division of Physiotherapy, Department of Neurobiology, Care Sciences and Society, Karolinska Institutet, 141 83 Stockholm, Sweden; 2 Allied Health Professionals Function, Functional area Occupational Therapy and Physiotherapy, Karolinska University Hospital, SE-171 76 Stockholm, Sweden; 3 Department of Women and Children’s Health, Karolinska Institutet, SE-171 76 Stockholm, Sweden

**Keywords:** Electromyography, Ergonomics, Kinematics, Kinetics, Manual handling, Movement analysis

## Abstract

**Background:**

Many workers performing manual handling tasks suffer from musculoskeletal disorders (MSD). Previous research has identified several loading aspects associated with manual handling, but it is still unknown if lifting on an unstable surface is associated with increased biomechanical loading of different body parts.

**Aim:**

This proof-of-concept study aims to study what kinematic and kinetic movement parameters, such as movement time, joint angles, torque, and muscle activity are feasible and of importance when studying the effect of lifting on surfaces with varying degrees of stability in an experimental set-up.

**Methods:**

Measurements were taken during three different surface conditions: stable, slightly unstable, and unstable. The participants were instructed to lift a box from the floor and place it on a table in front of them. The weight of the box varied from 0.5 to 15.5 kg. By using a motion capture system (VICON) with 28 reflective markers placed on the participants and one on the box, one Kistler force plate for measuring force levels and center of pressure movements (CoP), and four electromyographic transmitters (EMG), we analyzed the downward and upward phases of the lifting movement, using the Friedman’s test for repeated measures.

**Results:**

Statistically significant results with less joint movements in the lower and upper back were seen with increased instability during both the downward and upward phases. The decrease in trunk movements with increased instability resulted in a somewhat more flexed knee position during the movement, a lower torque in the lower back, and a decrease in CoP movements, but no differences in movement time or muscle activity in back and knee muscles.

**Conclusion:**

Lifting while standing on unstable surfaces resulted in an alteration of both kinematics and kinetics parameters; however, further studies regarding whether this is an additional risk factor for developing lower back pain are needed. Muscle activity levels were not altered due to instability and due to the complexity of the measurement, and we suggest not including EMG measures in future experiments of this type.

**Supplementary Information:**

The online version contains supplementary material available at 10.1186/s40814-022-01157-2.

## Key messages regarding feasibility

- Not many ergonomic studies have explored the importance of unstable surfaces as a risk factor for MSD, although in Sweden there are several regulations on this issue. The scientific operationalization of the concept of “instability” is still unknown. What movement parameters are of importance?
- The main findings from this proof-of-concept study showed that both kinematic and kinetic variables can differentiate between lifting on stable and unstable surfaces. However, EMG measures did not.
- These findings indicate that we should focus on these kinematic and kinetic variables and exclude measures of EMG in our planned main study.

## Introduction

A large proportion of the working population suffers from musculoskeletal disorders (MSD), e.g., back and knee pain [1–4]. One of the most important work-related risk factors for load-related MSD is manual handling in connection with twisted/bent lifting positions [5] and lifting from the floor [6]. Ergonomists have previously recognized that the object’s weight, shape, duration, and frequency [7], as well as lifting technique [8], are important risk factors since all these parameters are affecting the load levels of the musculoskeletal system. The Swedish Work Environment Authority, with the mission to secure the working environment of all workers in Sweden, recommend in their lifting recommendations that the surface the worker is standing on should be stable: “floors must be firm and stable, but should have “an elasticity” that is suitable for manual handling” [9]. Still, unstable lifting occurs in many professions such as loaders, farmers, forestry workers, military, and firefighters, but the importance of the stability of the surface for the development of MSD remains unclear.

Faber et al. tested whether the lifting technique was affected by a ship’s movements at sea and showed that the load levels increased [10], while Törner et al. showed that fishermen considered that their MSDs were related to the ship’s movements [11]. In a large study of the global burden of occupational diseases, workers in agriculture had three times greater risk of suffering from low back pain, and workers in the transport sector were found to have two times greater risk of suffering from low back pain when compared to other occupational categories [4]. Van Vuuren et al. showed that work on uneven or slippery surfaces tripled the risk of back pain in the South African steel industry [12, 13]. However, this association was tested with questionnaires instead of objective measurement methods since it is very difficult to quantify the degree of instability. It is difficult for the employer to implement the proposed recommendations, as there are no clear intended measures given in the regulations. There neither exist any objective methods for quantifying instability, which is a prerequisite for good risk analysis.

Human movement scientists analyze movements by studying three different aspects. At first, they will use “kinematics,” which concerns the study of temporal and spatial aspects of a movement such as movement time, linear and angular displacement, velocity, and acceleration of the body in space or body segments in relation to each other. Secondly, they also include the analysis of “kinetics,” which concerns the study of linear and angular forces that produce body movements but also the forces resulting from movements. Kinetics includes the analyses of the timing, direction, and magnitude of forces but also the point of application in relation to specific joints making it possible to estimate the biomechanical load on these joints. Finally, to fully understand the movements, most human movement scientists also include electromyographic (EMG) sensors applied on the skin above the muscle bellies that enable the study of force exertion and the timing aspects of muscles that are responsible for starting and controlling the movements.

In summary, previous research shows that manual handling of objects and people is important for the occurrence of MSD, and it seems that the unstable surfaces can increase muscle activity and joint load, but there is a lack of knowledge about both mechanisms and objective methods for quantifying risk levels. Basic knowledge is needed to be able to provide clearer load ergonomic recommendations for preventive measures in the field. As a first step, it is important to study the effect of unstable conditions on movement quality and biomechanical loading of different body segments in an experimental set-up. Therefore, it is crucial to study if kinematic, kinetic, and muscle activity parameters during manual handling of different weights are affected by different degrees of instability of the surface. This proof-of-concept study aims to study what kinematic and kinetic movement parameters, such as movement time, joint angles, torque, and muscle activity are feasible and of importance when studying the effect of lifting on surfaces with varying degrees of stability in an experimental set-up. Our hypothesis was that increased instability led to increased biomechanical loading of the knee and back joints.

## Methods

### Design

This is an experimental proof-of-concept study with a randomized approach. To minimize fatigue and learning factors, both surface condition and weight were randomized in a two-step process, using an online randomizer [14]. Firstly, we randomized the surface condition (stable, slightly unstable, and unstable) among the participants, so each surface condition was used as the first position approximately the same number of times. In the second step, the weights (0, 5, 10, and 15 kg) were then randomized for each surface condition.

### Participants

Seven participants took part in the study, two women and five men (Table 1). The participants were staff and students at the Division of Physical Therapy at the Karolinska Institutet and their personal contacts. The inclusion and exclusion criteria were designed to reflect a healthy and working population. Included were participants between the age of 18–65 years, with no ongoing MSD, and able to understand the lifting instructions. Prior to testing, the participants received written and oral information about the study, and informed consent was collected from all participants. They were also asked to fill in a questionnaire about demographics, training routines, and miscellaneous important information. Participants were asked to wear tight clothing, tied up hair, and be barefoot during the experiment. This study was conducted as a student project at the Karolinska Institutet and followed the guidelines from the Helsinki declaration of ethical principles (World Medical Association Declaration of Helsinki, 2013).

**Table 1:** Participants

**Age** median [year] (min–max)	**27** (21–47)	
**Sex** (male/female)	5/2	
**Ethnicity**	Swedish nationality	6
	Non-Swedish nationality	1
**Education** (lower/higher^a^)	0/7	
**Occupation**	Registered physical therapists	4
	Students	3
**Height** median [cm] (min-max)	**177** (163–185)	
**Weight** median [kg] (min-max)	**75** (69–85)	
**Physical activity** median [hours/week] (min–max)		
- Aerobic training	1 (0–10)	
- Strength training	2 (0.5–4.5)	
- Balance training	0 (0–1)	
Total minutes per week Mean (standard deviation)	317 (196)	

Higher educational level: at least 2 years of post-gymnasium studies

^a^Lower educational level: 2 or 3years of college

### Procedure

The measurements were performed using three surfaces with three different degrees of instability: stable, slightly unstable, and unstable. The stable surface consisted of an inbuilt force plate, which is perceived as an ordinary indoor floor. For the slightly unstable and unstable surfaces, a psoas-pillow (50 × 40 × 30 cm) was placed centered on the force plate and the participants were asked to stand on it. The psoas pillow has five firm sides and one softer side. During the “slightly unstable” lifts, the pillow was placed with the soft side up, which causes a slight compression under the feet that makes the situation somewhat wobbly, but relatively stable. During the “unstable” lifts, the soft side of the pillow was directed downwards, which was the most unstable situation. Before lifting on slightly unstable and unstable surfaces, the participants were asked to stand still on the pillow for at least 10 s before each trial to get acclimatized to the situation. These 10 s of normal standing compressed the pillow to the maximum and enabled standardization of the exact height. The participants did not receive any feedback on the movement quality or other parameters of their performance from the researchers.

### Task

**Fig. 1:**
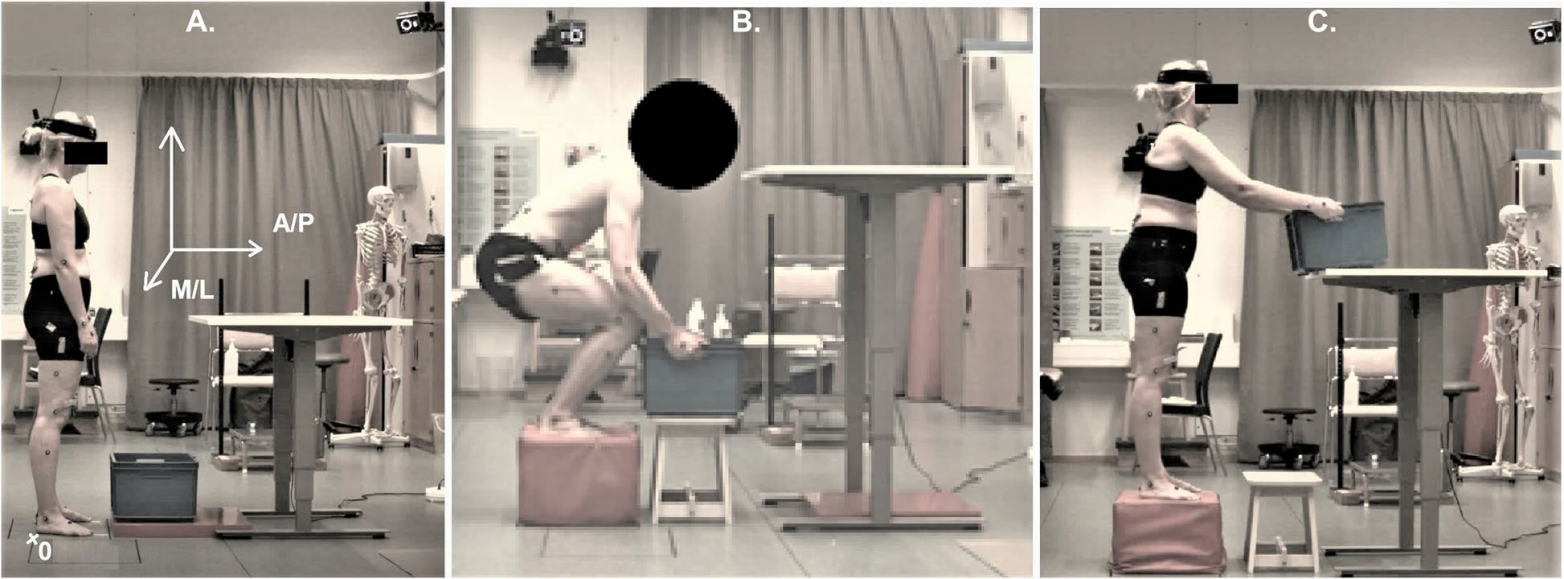
Experimental set-up (start, end of down-phase, end of up-phase). **a** Stable condition at the start position, **b** slightly unstable (soft side of the psoas pillow upwards) at end of down-phase/start of up-phase), and **c** unstable condition (soft side of the psoas pillow downwards) at end of up-phase. The origo (0) and coordinates system (M/L: medio-lateral; A/P: anterior-posterior) are put in **a**

The participants were given the task of lifting an object from just above ground level and placing it on a table in front of them. The object was a plastic box that weighed 0.495 kg, with the outer dimensions of 43 × 35 × 25 cm. To vary the lifting weight, extra weights of 5, 10, or 15 kg, in the form of dumbbells and weight cuffs, were placed firmly in the box. To standardize the test environment, the height of the table was adjusted to 50% of the participant’s length and the horizontal distance between the participant and the table was set to 75% of his/her arm length. Tape markings were used to ensure that all lifts were performed from and to the same position. When lifting on slightly unstable and unstable surfaces, the object was placed on a stool with a height exactly similar to the psoas pillow (see Fig. 1). The participants performed three consecutive lifts with about 10 seconds in between them, using the same weight while standing on the same surface. The first lift was used as a test trial and the two following were recorded. Between the lifts, the test staff returned the object to its original position, to avoid potential fatigue of the participants. In all situations, the third trial was used for the analyses to reduce a potential learning effect. In total, we analyzed three conditions and four weights (twelve lifts) for all seven participants, resulting in 28 × 3 repeated measures.

### Data collection

All data collection was done using an optical Motion capture system (Vicon, Oxford Metrics, UK) with 28 markers attached directly to the body using double-sided tape and a headband [12]. One additional marker was placed on the back of the box for identifying the movements of the box. These markers were used to create a 3D model of the subject (CGM1.1 - Vicon plugin gait), with a frequency of 100 Hz; hence, each frame corresponds to one centisecond (cs). Moreover, one Kistler platform (Winterthur, Switzerland) incorporated into the system was used to capture the ground reaction forces (1000 Hz). The participant was standing with both feet on the platform and all forces from the psoas pillow were captured since it fitted within the borders of the platform. Finally, electromyographic information (EMG) of muscles was measured with a wireless system (Noraxon, USA) using surface electrodes (Ag/AgCl, Blue Sensor N-00-S, Medicotest A/S, Ølstykke, Denmark) placed on the muscle bellies with an interelectrode distance of 20 mm (DE-02, size 23 mm × 17 mm), a bandwidth filtering of 0–500 Hz, and a sampling frequency of 1000 Hz. The signals were pre-amplified by factor 10. A reference electrode was placed near, but not on, the muscle belly being measured. EMG electrodes were attached bilaterally to the mm. Erector Spinae and above the muscle bellies of m vastus medialis, according to Seniam guidelines [15]. The same test leader attached the EMG electrodes to all participants. The participants were asked to contract the muscle and the respective abdominal muscles during palpation, and the electrodes were then secured with double-sided adhesive tape. Before attaching the electrodes, the skin was cleansed with isopropyl alcohol to reduce impedance from the skin, and the area was shaved if necessary, according to European recommendations [16]. The participants were asked to perform maximal voluntary contractions (MVC) of the two muscle groups. For measuring the MVC of the mm Erector Spinae, an isometric lumbar extension was performed, where the participant laid prone with the lower body on a bunk, with the hip ridge/SIAS against the edge so that the upper body hung outside. The participant was instructed to perform a maximum back lift, while the test leader then applied an adapted manual downward pressure on the participant’s shoulder blade while a research assistant stabilized the participant’s legs together with straps. For measuring the MVC of the knee extensor muscles, the participant was seated with a 90° hip and knee angles and pressing maximally with one foot at a time (right knee first) against a wall, while a research assistant stabilized the participant’s legs together with straps. The maximal pressure was maintained for at least 3 s.

### Data analysis

#### Kinematics

Each trial was divided into a downward phase and an upward phase. The start of the downward phase was defined as two frames before the speed of the C7 marker in the anterior/posterior (A/P) direction exceeded 2 mm/frame for three consecutive frames. The downward phase was ended upon the start of the upward phase, i.e., the first frame of three consecutive frames in which the box is moving upwards (vertical). The upward phase was terminated by the first of three consecutive frames showing that the box has stopped moving in the vertical direction. For each phase, foot, knee, hip, pelvic, spine, thorax, and neck angles were calculated in the sagittal plane (as provided by the software Vicon Nexus and model CGM1.1). Only the knee, hip, pelvic, lower, and upper back flexion angles were found to be of interest for answering the research questions. For these angles, we calculated the mean, the maximum, and minimum angles, and the difference between them defined then the total range of motion (ROM) of that joint for each movement phase. We calculated a mean of the left and right sides for all angles. A lower ROM of the back was interpreted as a lifting technique with a more upright position. We also divided this range by the number of frames for each phase for calculating a mean angular velocity for each joint.

#### Kinetics

The torque calculations from Vicon Nexus and model CGM1.1 were used to calculate the torque on the right hip, right knee, and lower back (L5) in the sagittal plane. We calculated the mean and maximal torque for both the downward and upward phases separately. Negative torque was interpreted as extensor torque.

**Fig. 2:**
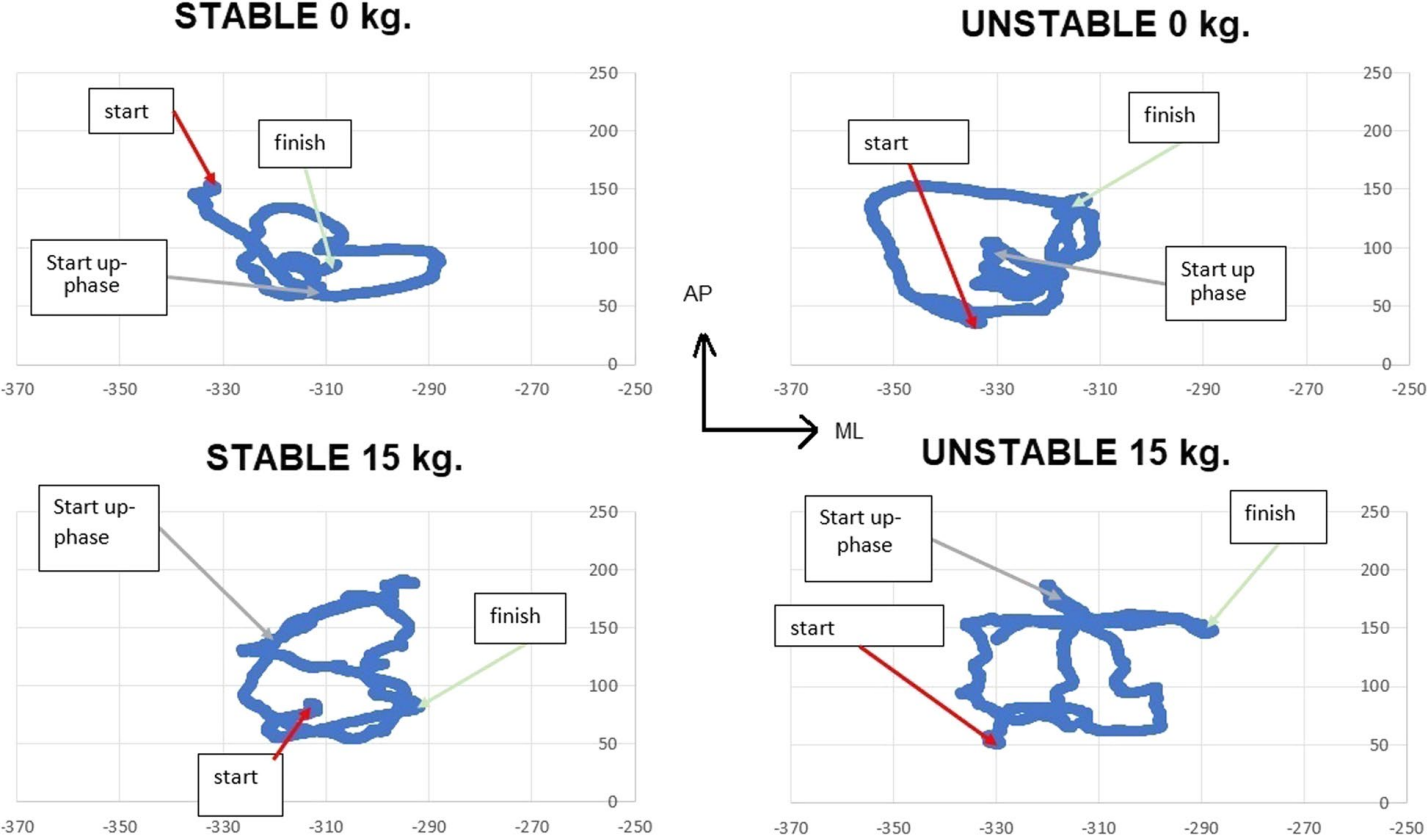
CoP point-to-point plot (mm). Movement in ML (*X*-axes, negative numbers) and AP direction (*Y*-axes, positive numbers) expressed in mm from the laboratory 0-point (see Fig. 1). The start of the downward phase is pointed with a red arrow, the start of the up-phase with a grey arrow, and the finish of the movement with a green arrow. For this individual, the heel markers were placed at around − 424 mm and toe markers (base of MP1) at around – 205 mm, showing that the CoP was kept in the midst of the base of support

Moreover, the center of pressure (COP) movements in the A/P and mediolateral (M/L) directions were calculated for each of the two phases separately. For each direction, the lowest (min–posterior) and highest (max–anterior) position was calculated for each phase. One typical example of a CoP point-to-point diagram for A/P and M/L movements during four different trials is found in Fig. 2. Finally, a total CoP movement was also calculated by taking the sum of all distances traveled for each different frame during the two phases and the whole movement, as calculated by using Pythagoras on A/P and M/L movements for each data point.

#### EMG

Raw EMG values were rectified, i.e., negative values of the EMG signal were transformed into positive values, by calculating the root mean square. These were then “smoothed” with a “moving windows method” of 50 data points. The EMG levels (mV) for each trial were then normalized by describing the EMG level as a percentage of the maximal muscle contractions (%MVC). Besides using the highest value (max), also the 95th percentile was used both for the MVC and the trials, to further reduce the effects of so-called artifacts. Finally, an average value (mean) for each phase was also calculated. All muscles were analyzed separately but also aggregated into one mean MVC of both the left and right sides for each muscle group. Sensitivity analyses using single muscles did not alter the results.

### Statistics

The downward and upward phase results for each variable of each trial were entered into a matrix using MS Excel for Windows 10 and exported to IBM SPSS Statistics version 27. Histograms were used to study if data were normally distributed using the aggregated data of all weights and all separate weights. Data were not found to be normally distributed, and therefore, non-parametric statistics were used: the median (min/max) was used for descriptive statistics and the Friedman’s test for repeated measures was used to calculate associations between the dependent (kinematic, kinetic, and EMG) variables, and the independent variable (surface) using all weights for each calculation. If significance occurred (*p* < 0.05), we examined between which conditions the possible differences could be found, using the Wilcoxon signed-rank test as a post hoc test, adjusting for multiple analyses with a Bonferroni correction. We also performed these analyses for each specific weight (0, 5, 10, and 15 kg) separately, but we decided to present the results from all weights together, since these contain four times more observations for each analysis. Using an online power calculator (http://powerandsamplesize.com/), the mean and standard deviation (SD) of all variables were used in a power analysis, *p* < 0.05, 80% power (1-β), to study the number of participants needed in the main study.

## Results

In total, 84 lifts were performed (7 subjects, 3 conditions, and 4 weights) without any adverse events or side effects. Data were available for all the ~100 variables in nearly all of the lifts. However, data concerning torque was lost in two lifts of one subject, while the knee EMG data during the four unstable conditions were considered erroneous in another subject.

### Kinematics

Table 2 shows the variables on movement time for the four weights together, but also for each weight separately. The time during the downward and upward phases did not differ between the different surfaces with weights of 10 kg or lower. However, there was a tendency to significance (Friedman’s test *p* = 0.085) that the time of the downward phase was shorter in the slightly unstable condition compared to the stable condition (unadjusted Wilcoxon paired test; *p* = 0.043). It was clear that an increased weight led to proportionally increased movement time in both phases.

**Table 2:** Temporal variables. Mean total movement time and time in the two phases in cent seconds (cs). Median (min, max) (*n* = 28)

**All weights**	**Stable**			**Slightly unstable**			**Unstable**			**Friedman**			
	**Median**	**Min**	**Max**	**Median**	**Min**	**Max**	**Median**	**Min**	**Max**	***p*-value**			
**Down**	102	72	132	102	76	134	100	80	156	0.260			
**Up**	150	82	171	135	89	171	142	84	172	0.552			
**Total**	249	166	300	233	166	305	234	165	328	0.104			
**0 kg.**	**Stable**			**Slightly unstable**			**Unstable**			**Friedman**			
	**Median**	**Min**	**Max**	**Median**	**Min**	**Max**	**Median**	**Min**	**Max**	***p*-value**			
**Down**	88	72	98	90	76	103	87	80	106	0.867			
**Up**	143	82	171	130	89	166	140	84	154	0.964			
**Total**	231	166	269	218	166	268	228	165	260	0.772			
**5 kg.**	**Stable**			**Slightly unstable**			**Unstable**			**Friedman**			
	**Median**	**Min**	**Max**	**Median**	**Min**	**Max**	**Median**	**Min**	**Max**	***p*-value**			
**Down**	102	98	113	101	91	134	100	93	114	0.565			
**Up**	142	94	171	144	100	171	132	100	149	0.630			
**Total**	251	202	269	235	195	305	232	193	263	0.317			
**10 kg.**	**Stable**			**Slightly unstable**			**Unstable**			**Friedman**			
	**Median**	**Min**	**Max**	**Median**	**Min**	**Max**	**Median**	**Min**	**Max**	***p*-value**			
**Down**	111	97	119	104	93	119	101	96	117	0.565			
**Up**	151	104	170	136	96	171	150	108	170	0.317			
**Total**	249	204	288	232	200	290	252	204	287	0.772			
**15 kg.**	**Stable**			**Slightly unstable**			**Unstable**			**Friedman**	**Wilcoxon Paired sign-rank (*p*-value)**		
	**Median**	**Min**	**Max**	**Median**	**Min**	**Max**	**Median**	**Min**	**Max**	**p-value**	**stable vs unstable**	**stable vs slightly unstable**	**slightly unstable vs unstable**
**Down**	120	99	132	104	91	126	111	99	156	0.085	0.500	**0.043**	1.000
**Up**	152	110	168	151	94	168	154	95	172	0.630			
**Total**	269	213	300	252	205	294	265	204	328	0.203			

Significant differences (*p* < 0.05) in bold

Table 3 shows the results from the joint angles for the different surfaces and all weights, while the results for the four weights separately are presented in a table in Additional file 1. Friedman’s test revealed differences between the conditions for several joints. Most interestingly, for the lower back, there was a significantly lower (md 5°) amount of ROM (the difference between min and max value) used during the downward phase of unstable lifting compared to stable lifting (Friedman’s test *p*-value 0.005; adjusted *p*-value 0.010), mainly because of a lower “maximal” angle that was used (Friedman’s test *p*-value 0.009; adjusted *p*-value 0.010). Around the same difference in ROM of the lower back (md 7°) between the stable and unstable lifting was seen also during the upward phase (Friedman’s test *p*-value 0.015; adjusted *p*-value 0.004), also mainly due to a difference in “maximal” angle (Friedman’s test *p*-value 0.002; adjusted *p*-value 0.004). Differences were also seen between the stable and slightly unstable situation and significant slower movements of the lower back during both the downward and upward phases (Friedman’s test *p*-value 0.002). The same pattern of decreased ROM was also seen in the upper back during both the downward and upward phases respectively (Friedman’s test *p*-value < 0.001 and 0.0009).

**Table 3:** Angles (°) and angular velocity (°/cs). Median (min, max) of different variables in the two movement phases (*n* = 28)

All weights	Stable			Slightly unstable			Unstable			Friedman	Wilcoxon Paired sign-rank (Bonferroni-adjusted)		
	Median	Min	Max	Median	Min	Max	Median	Min	Max	***p***-value	stable vs unstable	Stable vs slightly unstable	Slightly unstable vs unstable
**Hip**													
**Down**													
Mean	53.1	33.1	71.0	55.3	27.7	72.3	56.1	31.1	74.2	0.779			
Max	85.0	68.4	101.1	84.7	60.4	101.2	85.3	30.7	101.1	0.060			
Min	5.7	-3.0	67.2	6.4	-2.5	21.4	5.3	-2.1	23.1	0.565			
ROM	78.5	33.9	94.5	77.1	62.9	90.5	76.1	13.8	91.5	0.174			
Velocity	0.525	0.372	0.704	0.560	0.360	0.754	0.527	0.349	0.778	0.779			
**Up**													
Mean	39.9	30.1	61.2	40.0	27.2	62.8	40.1	24.7	72.4	0.898			
Max	84.3	68.2	99.4	84.1	60.4	99.9	85.6	62.5	99.8	0.174			
Min	14.4	5.8	29.8	14.5	4.9	31.4	13.2	-0.1	36.9	0.131			
ROM	71.0	55.7	81.1	69.9	53.6	76.7	71.7	54.3	87.4	0.565			
Velocity	0.282	0.177	0.600	0.296	0.166	0.655	0.309	0.160	0.763	0.779			
**Knee**													
**Down**													
Mean	52.5	25.1	78.3	51.0	24.0	78.6	51.5	27.9	76.7	0.215			
Max	93.2	39.7	114.2	90.7	38.9	112.2	89.4	36.8	111.6	0.629			
Min	8.7	-4.0	54.1	9.9	-3.4	17.9	11.7	-3.3	23.7	**0.007**	**0.002 (0.006)**	0.033 (0.098)	0.350 (1.000)
ROM	85.3	30.0	108.1	84.7	28.5	109.1	82.3	13.1	107.4	0.248			
Velocity	0.458	0.299	0.799	0.502	0.308	0.731	0.472	0.318	0.760	0.105			
**Up**													
Mean	29.0	18.9	51.8	28.6	15.5	47.6	32.9	18.0	61.7	0.409			
Max	90.7	39.5	106.9	89.7	38.9	108.0	94.6	43.7	110.3	0.779			
Min	10.9	-8.4	22.5	9.6	-7.2	18.9	11.4	-10.7	24.3	**0.004**	**0.016 (0.048)**	0.423 (1.000)	**0.001 (0.004)**
ROM	79.3	26.5	105.7	82.1	29.0	107.5	80.3	32.7	105.8	0.507			
Velocity	0.198	0.126	0.523	0.210	0.108	0.503	0.224	0.118	0.650	0.248			
**Pelvic**													
**Down**													
Mean	23.0	11.8	30.5	23.2	13.2	29.6	21.0	14.5	31.1	**0.018**	0.593 (1.000)	**0.008 (0.023)**	0.033 (0.098)
Max	33.8	15.4	42.6	31.8	18.5	41.4	32.2	9.7	43.1	**0.019**	0.109 (0.326)	**0.005 (0.015)**	0.229 (0.687)
Min	8.5	-0.7	33.4	8.8	-0.5	16.4	8.1	-3.5	19.2	0.629			
ROM	24.8	3.2	37.8	21.1	8.4	34.6	24.9	5.0	35.2	0.074			
Velocity	0.229	0.107	0.318	0.238	0.109	0.348	0.216	0.093	0.361	0.779			
**Up**													
Mean	22.2	11.2	30.7	21.2	11.7	33.5	21.6	8.0	32.9	0.507			
Max	35.7	19.9	42.3	33.0	20.2	41.9	34.0	21.1	41.7	**0.050**	0.109 (0.326)	**0.016 (0.048)**	0.423 (1.000)
Min	13.4	4.0	20.9	13.2	4.6	23.8	12.8	-4.4	24.3	0.507			
ROM	19.3	13.8	30.3	19.1	13.0	26.6	22.2	13.2	33.3	0.156			
Velocity	0.162	0.066	0.302	0.165	0.069	0.349	0.184	0.046	0.367	0.779			
**Lower back**													
**Down**													
Mean	16.1	3.5	43.9	14.7	5.2	42.0	16.1	-3.4	39.9	0.368			
Max	31.1	18.5	62.7	30.0	15.8	58.9	30.6	14.6	60.0	**0.009**	**0.003 (0.010)**	0.023 (0.0069)	0.504 (1.000)
Min	-7.7	-15.8	4.1	-9.3	-15.3	4.5	-7.3	-21.3	22.0	0.965			
ROM	41.2	16.0	60.9	39.7	26.4	57.3	36.2	4.4	60.1	**0.005**	**0.003 (0.010)**	**0.008 (0.023)**	0.789 (1.000)
Velocity	0.151	0.027	0.503	0.151	0.062	0.478	0.146	-0.035	0.493	**0.009**	0.023 (0.069)	**0.003 (0.010)**	0.504 (1.000)
**Up**													
Mean	8.5	1.1	43.6	6.5	-2.9	41.2	8.1	-0.8	38.8	**<0.001**	0.350 (1.000)	**0.003 (0.010)**	**<0.001 (<0.001)**
Max	30.6	18.2	62.7	29.9	15.6	58.9	30.2	14.5	59.9	**0.002**	**0.001 (0.004)**	**0.005 (0.015)**	0.688 (1.000)
Min	-4.6	-11.8	19.6	-6.2	-12.5	18.9	-4.9	-14.9	18.6	**0.005**	0.593 (1.000)	**0.002 (0.006)**	**0.011 (0.033)**
ROM	38.0	26.5	46.3	35.9	27.4	46.9	31.9	25.9	48.4	**0.015**	**0.005 (0.015)**	0.423 (1.000)	0.045 (0.135)
Velocity	0.053	0.007	0.532	0.047	-0.022	0.412	0.072	-0.009	0.457	**0.002**	0.350 (1.0)	**0.011 (0.033)**	**0.001 (0.002)**
**Upper back**													
**Down**													
Mean	-41.1	-63.9	-21.2	-39.4	-60.1	-29.6	-39.9	-59.7	-17.1	**0.050**	**0.016 (0.048)**	0.109 (0.326)	0.432 (1.000)
Max	-0.8	-7.1	3.0	-1.4	-9.1	3.3	-1.5	-26.5	5.3	0.331			
Min	-64.4	-98.7	-45.5	-61.3	-92.0	-43.1	-59.5	-92.4	-3.3	**<0.001**	**0.002 (0.006)**	**<0.001 (0.001)**	0.504 (1.000)
ROM	63.1	45.2	96.5	59.4	44.6	89.9	57.0	6.1	91.1	**<0.001**	**0.001 (0.004)**	**<0.001 (0.001)**	0.688 (1.000)
Velocity	-0.411	-0.699	-0.166	-0.395	-0.650	-0.249	-0.383	-0.674	-0.174	**0.039**	0.033 (0.098)	0.023 (0.069)	0.894 (1.000)
**Up**													
Mean	-31.9	-64.3	-19.4	-30.8	-61.4	-21.2	-31.3	-61.2	-25.3	**0.039**	0.285 (0.855)	**0.011 (0.033)**	0.142 (0.425)
Max	-11.3	-30.1	-2.8	-10.2	-28.7	-1.4	-9.6	-27.6	-2.7	0.331			
Min	-64.2	-98.6	-46.8	-60.1	-91.7	-46.6	-59.9	-92.3	-45.0	**<0.001**	**0.002 (0.006)**	**<0.001 (0.001)**	0.142 (0.425)
ROM	51.7	36.1	73.8	50.4	38.1	70.4	51.5	35.4	70.6	**0.009**	0.023 (0.069)	**0.003 (0.010)**	0.504 (1.000)
Velocity	-0.220	-0.783	-0.116	-0.211	-0.614	-0.128	-0.226	-0.670	-0.171	0.055			

Significant differences (*p* < 0.05) in bold

Velocity expressed in °/cs

At the same time, the knees remained more flexed (a higher “min” degree) during the unstable condition during the downward and upward phases respectively (Friedman’s test *p*-value 0.007 and 0.004; adjusted *p*-value 0.006 and 0.048), compared to the stable condition. Moreover, during the stable condition, the maximal pelvic tilt was larger compared to the slightly unstable position, both during the downward and upward phases (Friedman’s test *p*-value 0.019 and 0.050; adjusted *p*-value 0.015 and 0.048).

### Kinetics

Table 4 presents the torque calculations for the three joints of interest. We lost data for the hip and lower back for one subject during the 5 kg and 10 kg unstable trials. Friedman’s tests revealed a lower maximal back-torque in the down-phase during the unstable condition (md 776.5 Nmm) when compared with stable (md 849.0 Nmm) and slightly unstable (821.6 Nmm) conditions (Friedman’s test *p*-value 0.015; adjusted *p*-value 0.050 and 0.025, respectively). The mean torque around the knee during the downward phase was also the lowest in the unstable (216.8 Nmm) condition (Friedman’s test *p*-value 0.012; adjusted *p*-value 0.010) compared to the slightly unstable (280.6 Nmm) condition. In the upward phase, a lower maximum torque around the knee during the unstable (320.7 Nmm) condition was found (Friedman’s test *p*-value 0.005; adjusted *p*-value 0.004) compared to the stable (359.5 Nmm) condition.

**Table 4:** Mean and maximal torque (Nmm) on hip, knee, and back joints during the two movement phases. Median (min, max) (*n* = 28)

	Stable			Slightly unstable			Unstable			Friedman	Wilcoxon Paired sign-rank (Bonferroni-adjusted)		
	Median	Min	Max	Median	Min	Max	Median	Min	Max	***p***-value	stable vs unstable	stable vs slightly unstable	slightly unstable vs unstable
**Hip**													
Down													
Mean	543.55	152.3	731.0	536.6	114.2	703.3	522.1^a^	121.45^a^	703.15^a^	0.607			
Max	1227.1	468.65	1866.25	1183.95	470.0	1797.35	1125.0^a^	469.7^a^	1799.5^a^	0.304			
Up													
Mean	558.6	67.1	1175.4	553.85	67.25	1193.3	517.5^a^	101.75^a^	1135.25^a^	0.304			
Max	1379.3	522.9	2096.05	1392.7	469.7	2129.05	1260.7^a^	457.65^a^	1889.7^a^	0.63			
**Knee**													
Down													
Mean	230.0	-57.7	761.65	280.55	-18.0	619.6	216.75	-3.9	591.5	**0.012**	0.061 (0.184)	0.285 (0.855)	**0.003 (0.010)**
Max	442.0	208.0	1125.65	449.15	261.15	1027.4	398.15	253.65	895.7	0.131			
Up													
Mean	67.0	-467.35	360.35	62.7	-396.8	296.55	-48.8	-437.65	405.7	0.113			
Max	359.5	-165.45	1191.65	365.85	-210.75	953.6	320.7	-211.05	890.5	**0.005**	**0.001 (0.004)**	0.229 (0.229)	0.045 (0.135)
**Lower back**													
Down													
Mean	627.3	158.8	994.7	683.9	120.9	950.7	625.3^a^	131.6^a^	1065.2^a^	0.446			
Max	1698.0	658.9	2611.5	1643.1	713.5	2581.9	1552.9^a^	737.7^a^	2591.7^a^	**0.015**	**0.018 (0.050)**	**0.008 (0.025)**	0.782 (1.000)
Up													
Mean	862.0	61.0	1875.7	836.5	33.5	1942.8	808.5^a^	83.2^a^	1795.3^a^	0.248			
Max	1988.0	773.0	3045.6	1904.2	737.3	3226.5	1811.3^a^	683.9^a^	2775.9^a^	0.06			

Significant differences (*p* < 0.05) in bold

Torque expressed in Nmm: positive numbers = flexion, negative numbers = extension

^a^*n* = 26

The variables regarding the center of pressure measures can be found in Table 5, while the data for the separate weights can be found in a table in Additional file 2. There was a decreased mean and maximal displacement in the AP direction in the unstable condition during the upward phase compared to the stable condition (Friedman’s test *p*-value < 0.001 and 0.002; adjusted *p*-value < 0.001 and 0.023, respectively). Interestingly, there were significantly more movements in the AP direction in the downward phase in the slightly unstable condition for the minimal and total sum, compared to the stable condition (Friedman’s test *p*-value 0.017 and 0.002; adjusted *p*-value 0.023 and 0.006, respectively). This was also seen in the M/L direction, where this difference was most profound in the variable maximal position for both the downward and upward phases (Friedman’s test *p*-value 0.048 and 0.012; adjusted *p*-value 0.042 and 0.010, respectively).

**Table 5:** Center of pressure (CoP) variables in anterior/posterior (A/P) and mediolateral (M/L) direction during the two movement phases. Median (min, max) (*n* = 28)

	Stable			Slightly unstable			Unstable			Friedman	Wilcoxon Paired sign-rank (Bonferroni-adjusted)		
	Med	Min	Max	Med	Min	Max	Med	Min	Max	***p***-value	stable vs unstable	stable vs slightly unstable	slightly unstable vs unstable
**Down**													
**A/P Mean displacement**	0.074	-0.015	0.122	0.064	0.003	0.108	0.074	0.003	0.111	0.897			
**A/P Min position**	52.99	25.69	85.89	68.90	27.33	117.68	56.61	36.70	88.21	**0.017**	0.539 (1.000)	**0.008 (0.023)**	0.033 (0.098)
**A/P Max position**	137.88	90.66	188.15	144.72	104.91	185.92	143.73	98.48	191.23	0.101			
**A/P Diference min/max**	82.74	40.63	134.69	84.59	26.73	121.98	86.00	34.63	124.63	0.775			
**A/P Total sum**	227	137	360	267	119	382	231	143	440	**0.002**	0.894 (1.000)	**0.002 (0.006)**	**0.003 (0.019)**
**Up**													
**A/P Mean displacement**	0.021	-0.011	0.044	0.007	-0.096	0.059	-0.001	-0.071	0.034	**<0.001**	**<0.001 (<0.001)**	**0.001 (0.004)**	0.229 (0.687)
**A/P Min position**	53.19	5.00	125.33	55.04	32.69	101.96	49.76	17.51	83.22	0.501			
**A/P Max position**	169.96	91.85	209.00	167.67	117.51	219.98	154.77	103.89	190.80	**0.002**	**0.008 (0.023)**	0.504 (1.000)	**0.001 (0.003)**
**A/P Diference min/max**	122.33	39.20	166.76	116.77	55.43	160.98	101.65	34.53	166.61	0.501			
**A/P Total sum**	352	176	533	393	145	475	309	155	506	0.501			
**Down**													
**M/L Mean displacement**	-0.001	-0.025	0.035	-0.001	-0.033	0.023	0.001	-0.030	0.033	0.623			
**M/L Min position**	-329.92	-354.58	-311.29	-324.75	-344.33	-312.13	-338.13	-357.49	-298.28	0.105			
**M/L Max position**	-296.52	-322.99	-275.43	-293.44	-315.42	-270.95	-304.83	-327.64	-269.81	**0.048**	0.304 (0.913)	0.153 (0.458)	**0.014 (0.042)**
**M/L Difference min/max**	30.08	17.43	62.33	32.91	7.19	56.26	27.72	11.98	69.77	0.101			
**M/L Total sum**	255	171	396	253	159	400	256	173	462	0.623			
**Up**													
**M/L Mean displacement**	0.00	-0.03	0.04	0.000	-0.030	0.029	0.009	-0.027	0.026	0.324			
**M/L Min position**	-329.11	-374.72	-315.48	-323.61	-361.19	-295.37	-334.49	-375.56	-297.49	0.087			
**M/L Max position**	-294.41	-339.88	-263.22	-288.04	-321.08	-256.18	-302.94	-336.61	-250.78	**0.012**	0.142 (0.425)	0.142 (0.425)	**0.003 (0.010)**
**M/L Difference min/max**	37.21	15.06	56.81	38.35	16.71	67.83	35.40	14.69	64.63	0.646			
**M/L Total sum**	276	194	512	340	142	497	287	197	423	0.324			
**Down**													
**Total displacement Mean**	0.401	0.290	0.494	0.420	0.290	0.516	0.376	0.211	0.465	0.083			
**Total displacement total sum**	0	0	0	411	221	608	383	247	641	0.137			
**Up**													
**Total displacement Mean**	0.008	0.000	0.020	0.405	0.290	0.444	0.360	0.243	0.467	0.212			
**Total displacement Total sum**	490	306	789	586	224	741	485	278	764	0.593			
**Down**													
**COP area**	2233	970	7862	2361	488	5321	2167	865	7357	0.721			
**Up**													
**COP area**	4588	1205	7302	4600	936	8088	2910	1276	8607	0.623			

Significant differences (*p* < 0.05) in bold

Displacement in mm

COP area in mm^2^

### EMG

For one subject, the EMG recordings revealed in %MVC > 100% for the knee muscles during the four unstable trials, and these trials were excluded. There were no differences in EMG levels between the different surfaces (Table 6), even when analyzing the weights separately. The EMG recordings showed increased activity with increased weight, but not with increased instability.

**Table 6:** EMG (%MVC) variables of knee and back muscles during the two movement phases. Median (min, max) (*n* = 28 or *n* = 24^a^)

	Stable			Slightly unstable			Unstable			Friedman
	Median	Min	Max	Median	Min	Max	Median	Min	Max	*p*-value
**Knee**										
Down										
Mean	10.1	3.3	20.6	10.6	4.1	19.9	11.4	4.1	19.4	0.131
95p	33.8	9.6	80.6	37.1	11.4	67.4	36.5	11.9	67.7	0.368
Max	17.1	8.2	75.3	16.2	8.6	40.1	19.1^a^	9.1^a^	40.2^a^	0.687
Up										
Mean	6.8	2.4	14.9	6.7	3.1	26.9	6.8	1.6	14.7	0.867
95p	26.7	7.2	45.2	26.2	10.1	73.6	28.7	6.1	71.9	0.630
Max	12.8	6.4	81.1	12.5	8.1	32.3	12.7^a^	6.7^a^	28.1^a^	0.582
**Lower back**										
Down										
Mean	8.4	3.9	16.3	8.1	4.2	22.6	8.6	5.0	15.0	0.629
95p	27.9	13.7	49.3	26.3	13.4	62.2	26.2	14.0	53.4	0.779
Max	14.3	6.7	37.2	14.6	6.6	33.1	13.9	6.8	29.8	0.629
Up										
Mean	11.0	4.9	23.1	12.2	4.3	19.9	10.2	5.1	20.4	0.651
95p	31.3	13.5	67.5	32.8	12.0	55.4	27.7	14.5	63.2	0.867
Max	15.5	6.6	34.0	17.7	5.9	33.9	16.3	7.1	40.0	0.472

All values in %MVC

^a^*n* = 24

### Power

Based on this small sample, the results showed that the above-mentioned kinematic and kinetic parameters were significantly influenced by the degree of instability when using the data of all weights together. The power calculations revealed that if 30 participants are included in the main study, significant differences could be detected in 52 of the included variables (47.7%). If using 20 participants, 41 variables (37.6%) reach enough power for the analyses, while this number increases to 57 (52.3%) if 40 participants are going to be included. However, using 30 participants does not give enough power to detect differences in EMG levels in any of the variables, except for one variable in the upward direction.

## Discussion

The results from this proof-of-concept study revealed changes in lifting technique toward a more upright position when standing on an unstable surface. Against our hypothesis, we found that increased instability resulted in a somewhat lower range of movement in the lower and upper back, a lower torque around both knee and lower back, but an increased knee flexion during the movement. The participants seem to use a lifting strategy in which the movements of the center of pressure (reflecting the body position) decrease with increased instability. However, these differences did not result in any changes in muscle activity. Muscle activity levels do not seem to be altered due to instability, and because of the complexity of the measurement, we suggest not including EMG measures in future experiments of this type. This proof-of-concept study enabled us to calculate the number of subjects needed for each variable.

### Discussion of the main findings

This proof-of-concept study showed that the experimental setup is feasible for studying kinetic and kinematic variables of interest. Both the downward and upward movements during unstable conditions showed differences from stable lifting. Around 30 participants performing 4 lifting tasks in three different degrees of instability seems to be appropriate to be able to detect significant differences in 50% of the variables. Increasing to 40 participants does not change this proportion to a large extent. We believe that the degree of instability used in the experiment corresponds well to the instability occurring in different professions with manual handling; however, we should increase the external validity of the future experiment by including participants that perform lifting tasks in their profession.

Concerning the results, we believe that our results are in line with previous studies showing that lifting on unstable surfaces alters the lifting technique [10–13]. Our results can be described as with increased instability there is a shift towards a more vertical (upright) back position to keep the center of mass within the base of support with a larger marginal, by using more flexion in the knees. In theory, both the front side (quadriceps) and the back side of the thigh (hamstrings and gluteal muscles) should compensate for this larger use of the knee joints, while the back muscles mainly have a stabilizing function and a decrease in muscle activity [17]. During the stable lifts, the lift was performed with a larger ROM in the lower and upper back with, theoretically, a lower activation of the gluteal muscles [18]. Our results pointed in this direction; we saw an increased knee muscle activity in the upward phase, together with a decreased back muscle activity. We did not study the hamstrings and gluteal muscles and the activation of these muscles could be of interest in further studies. For example, Zemkova and Marshall et al. showed that muscle activation levels were higher both in a squat and during exercising with a dumbbell press on an unstable compared to a stable surface [19–21]. However, the methods used in these studies differed largely from our study. In these studies, several additional muscles were tested and a greater load (between 60% of 1RM and 6RM) was used. In the squat study the average load was 137 + − 28kg and in the dumbbell press study 20.62 + − 7.22kg [19, 20]. This was heavier than the load in our study where 15 kilos was the heaviest weight. The 15 kg limit was chosen since it is the maximal recommended weight by the Swedish Work Environment Authority during a working situation in which the weight is held more than ¾ arm length [9]. To standardize the degree of instability between studies is very difficult, which makes a comparison between studies challenging.

The results from our sensitivity analyses showed that movement time, segmental angles, torque, and back and knee EMG levels increased in relation to the weight of the object. This shows that increased load seems to be a more important factor than increased instability, but these results have been shown previously [22] and was not the focus of the present study.

### Methodological considerations

The use of a laboratory environment has both advantages and disadvantages. Previous field studies have shown that manual handling and work on unstable surfaces lead to an increased risk of developing low back pain [11–13]. In a laboratory environment, concurrent important risk factors linked to the risk of developing such disorders, such as psychosocial factors and stress, are difficult to be studied. Moreover, the increased risk of accidents or falls could be studied since the experiments were (for ethical reasons) performed under safe conditions. The experimental setup, on the other hand, allowed us to study many biomechanical aspects at the same time using highly valid methods, compared to field studies. The EMG measurements could also be a source of error, as the amount of subcutaneous fat, as well as the skin’s characteristics, can contribute to reduced EMG signals [23], and the use of MVC could lead to under- or overestimation of the activation levels and large individual variations.

In our study, students and staff members from the university were included. We believe that the main study should include workers with experience in unstable lifting, e.g., fishermen, farmers, truck drivers, scaffolders, etc., to have a possibility to extrapolate the results to the population of interest. At the time of measurement, these workers should be pain-free, since we know from previous studies that ongoing pain could alter lifting techniques [24–26]. It is, however, difficult to estimate what the impact of the choice of these subjects could have on the results in the main study. We know that experience and expectations can alter motor control strategies [26] and a previous experience of unstable floors during heavy lifting could have led to the development of a specific lifting technique. For example, the workers could have expectations that there could be disturbances of the base of support during their lifting task, and they have developed one specific lifting technique that applies to possible situations: stable, semi-instable, and unstable conditions [24]. This could lead to lesser differences between the different conditions. On the other hand, the worker could also have developed different movement schemes for different lifting conditions due to the exposure to many different lifting situations (and/or low back pain) during their career [26], and they might have developed an ability to instantly choose an appropriate lifting technique for each situation. This could then result in larger differences between the different lifting conditions in the experiment. We believe therefore that we should expect that by including pain-free workers in the main study, there could be differences in the results when using students and staff as in our experiment.

Although research indicates that lumbar spine problems are as prevalent in men as in women, it should be mentioned as a shortcoming that it was not possible to obtain an even distribution between the sexes, as there are differences between the sexes in muscle mass as well as biometric and biomechanical differences. In this study, however, the distances between the participants and the table as well as the height of the table were standardized to the individual, which should have given comparable test conditions for all participants. We did not study the effect on the shoulder joints, since a more upright position during lifting could, in theory, also increase the biomechanical loading of the shoulder muscles. The use of the mean of the left and right sides in our analyses could have diluted potential differences due to asymmetric lifting when standing on an unstable surface, and we plan therefore to analyze the dominant and non-dominant sides separately in the main study. On the other hand, this could also have omitted different artifacts and lowered the number of missing data. Finally, we used a psoas pillow to simulate slightly unstable and unstable surfaces, which could question the generalizability of a working situation. The psoas pillow certainly fulfilled the task of creating an unstable surface, but to what extent this can be equated with other unstable surfaces, such as a boat on the water, or lifting bricks on a scaffold remains unclear. Although some balance was required to remain still on the psoas pillow, there was a low risk for the participants to lose their balance, which we felt was important for safety reasons. In our main study, we plan to use a BOSU-up and BOSU-down approach as in the study of Busca et al. [15]. For further studies of unstable surfaces, we believe that researchers should study surfaces with higher demands on balance, for example, a larger gym mat, or tests in an authentic work environment, such as on a boat.

### Future studies

This study suggests that one should continue to study, in larger studies on 30 participants with working experience of unstable lifting conditions, whether there is an increased biomechanical joint load during unstable surfaces, and if asymmetric lifting occurs. The proof-of-concept study showed interesting results on kinematic and kinetic features; however, muscle activity levels were not altered, and due to the complexity of the measurement, we suggest not including EMG measures in future experiments of this type. Continuing this research with field studies in workplaces where lifting on unstable surfaces occurs would also be of interest, as it would be more comparable to the epidemiological studies that try to link unstable surfaces with MSP, such as low back pain or knee pain.

## Conclusion

Lifting while standing on unstable surfaces resulted in a change of movement kinematics and kinetics; however, further studies on a working population with experience of unstable lifting conditions regarding whether this is a significant risk factor for MSD are needed. Muscle activity levels were not altered due to instability, and due to the complexity of the measurement, we suggest not including EMG measures in future experiments of this type.

## Supplementary Information

**Additional file 1. **Angles for the different weights (0-15 kg) and lifting conditions (stable, slightly unstable, unstable) during the downward and upward phases (*n* = 7).

**Additional file 2. **CoP variables for the different weights (0-15 kg) and lifting conditions stable, slightly unstable, unstable) during the downward and upward phases (*n* = 7).

## Data Availability

The datasets generated during and/or analyzed during the current study are available from the corresponding author on reasonable request.
