# Supplementary material for: Biomechanical analysis of lifting on stable versus unstable surfaces—a laboratory-based proof-of-concept study
Source: Pilot Feasibility Stud. 2022 Sep 8;8:200. doi: 10.1186/s40814-022-01157-2 (PMC9454131; doi:10.1186/s40814-022-01157-2)
Supplement: Supplementary file 2 — Additional file 2. CoP variables for the different weights (0-15 kg) and lifting conditions stable, slightly unstable, unstable) during the downward and upward phases (n = 7). [file 40814_2022_1157_MOESM2_ESM.docx]

**Additional file 2. CoP variables for the different weights and lifting conditions during the down- and up-phases (n=7).**
